# The tumor suppressive role of miRNA-370 by targeting FoxM1 in acute myeloid leukemia

**DOI:** 10.1186/1476-4598-11-56

**Published:** 2012-08-17

**Authors:** Xiaolu Zhang, Jiping Zeng, Minran Zhou, Bingnan Li, Yuanyuan Zhang, Tao Huang, Lixiang Wang, Jihui Jia, Chunyan Chen

**Affiliations:** 1 Department of Hematology, Qilu Hospital, Shandong University, No.107, Wenhua Xi Road, Jinan, 250012, Shandong, P. R. China; 2 Department of Biochemistry, Shandong University, Jinan, China; 3 Department of Microbiology/Key Laboratory for Experimental Teratology of Chinese Ministry of Education, School of Medicine, Shandong University, Jinan, China; 4 Department of Medicine, Division of Hematology and CMM, Karolinska University Hospital Solna and Karolinska Institutet, Stockholm, Sweden

**Keywords:** miR-370, FoxM1, AML, Cellular senescence

## Abstract

**Background:**

Recent evidence has accumulated that MicroRNA (miRNA) dysregulation occurs in the majority of human malignancies including acute myeloid leukemia (AML) and may contribute to onco-/leukemo-genesis.

**Methods:**

The expression levels of miR-370 and FoxM1 were assessed in 48 newly diagnosed AML patients, 40 AML patients in 1^st^ complete remission (CR) and 21 healthy controls. Quantitative real-time PCR, western blots, colony formation assay, and β-Galactosidase ( SA**-**β-Gal) staining were used to characterize the changes induced by overexpression or inhibition of miR-370 or FoxM1.

**Results:**

We found that the down-regulation of miR-370 expression was a frequent event in both leukemia cell lines and primary leukemic cells from patients with de novo AML. Lower levels of miR-370 expression were found in 37 of 48 leukemic samples from AML patients compared to those in bone marrow cells derived from healthy adult individuals. Ectopic expression of miR-370 in HL60 and K562 cells led to cell growth arrest and senescence. In contrast, depletion of miR-370 expression using RNA interference enhanced the proliferation of those leukemic cells. Mechanistically, miR-370 targets the transcription factor FoxM1, a well established oncogenic factor promoting cell cycle progression. Moreover, when HL60 and K562 cells were treated with 5-aza-2^′^-deoxycytidine, a DNA methylation inhibitor, miR-370 expression was up-regulated, which indicates epigenetic silencing of miR-370 in leukemic cells.

**Conclusions:**

Taken together, miR-370 may function as a tumor suppressor by targeting FoxM1, and the epigenetic silence of miR-370 thus leads to derepression of FoxM1 expression and consequently contributes to AML development and progression.

## Introduction

Acute myeloid leukemia (AML) is a heterogeneous group of neoplastic haematopoietic diseases characterized by proliferation and maturation arrest of myeloid blasts in bone marrow and blood [1]. The long-term overall survival (OS) rate for AML patients under the age of 60 years and 60 years or older is 30–40% and under 10%, respectively [2], which remains a challenge. Thus, it is urgently needed to search for new targets for molecularly designed therapies.

microRNAs (miRNAs), small (~22 nucleotide), single-stranded noncoding RNAs, are a novel class of biological molecules. Their genes may either give rise to single miRNAs, or contain several miRNAs in one transcriptional unit as miRNA clusters [3]. miRNAs post-transcriptionally repress gene expression by recognizing complementary target sites in the 3^′^untranslated region (UTR) of target mRNAs [4,5]. miRNAs have been implicated in a large variety of biological processes, including cell cycle progression, apoptosis, differentiation and haematopoiesis [6-10], and thereby play important roles in many pathological processes, including malignant transformation [11,12]. More than 50% of miRNA genes are located in cancer-associated genomic regions or in fragile sites, and both oncogenic and tumor suppressive functions have thus far been ascribed to specific miRNAs [13]. Moreover, miRNAs have emerged as critical regulators of hematopoiesis and their aberrant expression has been associated with the pathogenesis of leukemia [14]. Functional validation of deregulated miRNAs in hematopoeisis has been shown for several miRNAs [15]. Distinctive patterns of increased expression and/or silencing of multiple miRNAs have been associated with specific cytogenetic and molecular subsets of AML [16]. miR-370 has been noted to be down-regulated in papillary thyroid carcinoma, colorectal cancer [17] and malignant cholangiocytes [18], but evidence of a biological role for this miRNA in AML has not been reported. In the present study, we sought to define the role of miR-370 in AML by investigating its expression and biological function in leukemic cell lines and blast cells from patients with de novo AML.

## Results

### Down-regulation of miR-370 expression in BM blasts from de novo AML patients

**Figure 1:**
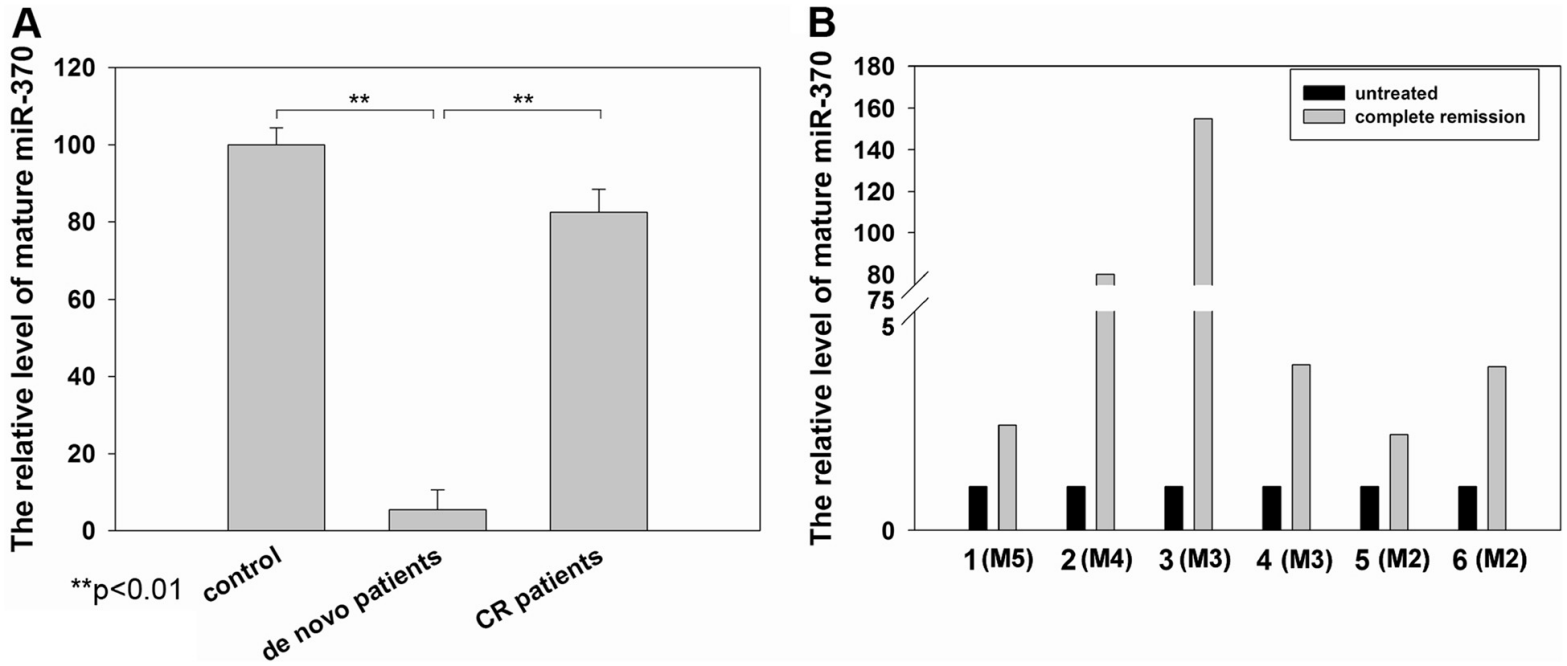
**Downexpression of miR-370 in de novo AML patients.** (**A**) *miR-370* expression by qRT-PCR in 48 de novo AML patients, 40 AML patients of 1^st^ CR and 21 healthy controls. (**B**) Restoration of miR-370 expression in six patients after complete remission achievement in induction chemotherapy. The results are shown as miRNA expression after normalization with U6 and -ΔCt calculations

We analyzed miR-370 expression in BM samples from 48 de novo AML patients at diagnosis time using qRT-PCR. As shown in Figure 1A, the miR-370 level in patients’ samples was significantly reduced (P < 0.01, t test) compared to that from healthy controls, while following acquisition of CR in the induction chemotherapy, miR-370 expression level restored to 0.82 fold of controls. There was no association between the presence of mature miR-370 and age, gender, blast percentage or FAB subtypes (data not shown). In six patients, BM samples were available both at diagnosis time prior to treatment and after a complete remission and we found a lower miR-370 level at diagnosis while at least 2.1-fold increase in miR-370 expression after CR (Figure 1B).

### Changes in proliferation and cellular senescence of leukemic cells mediated by altered miR-370 expression

**Figure 2:**
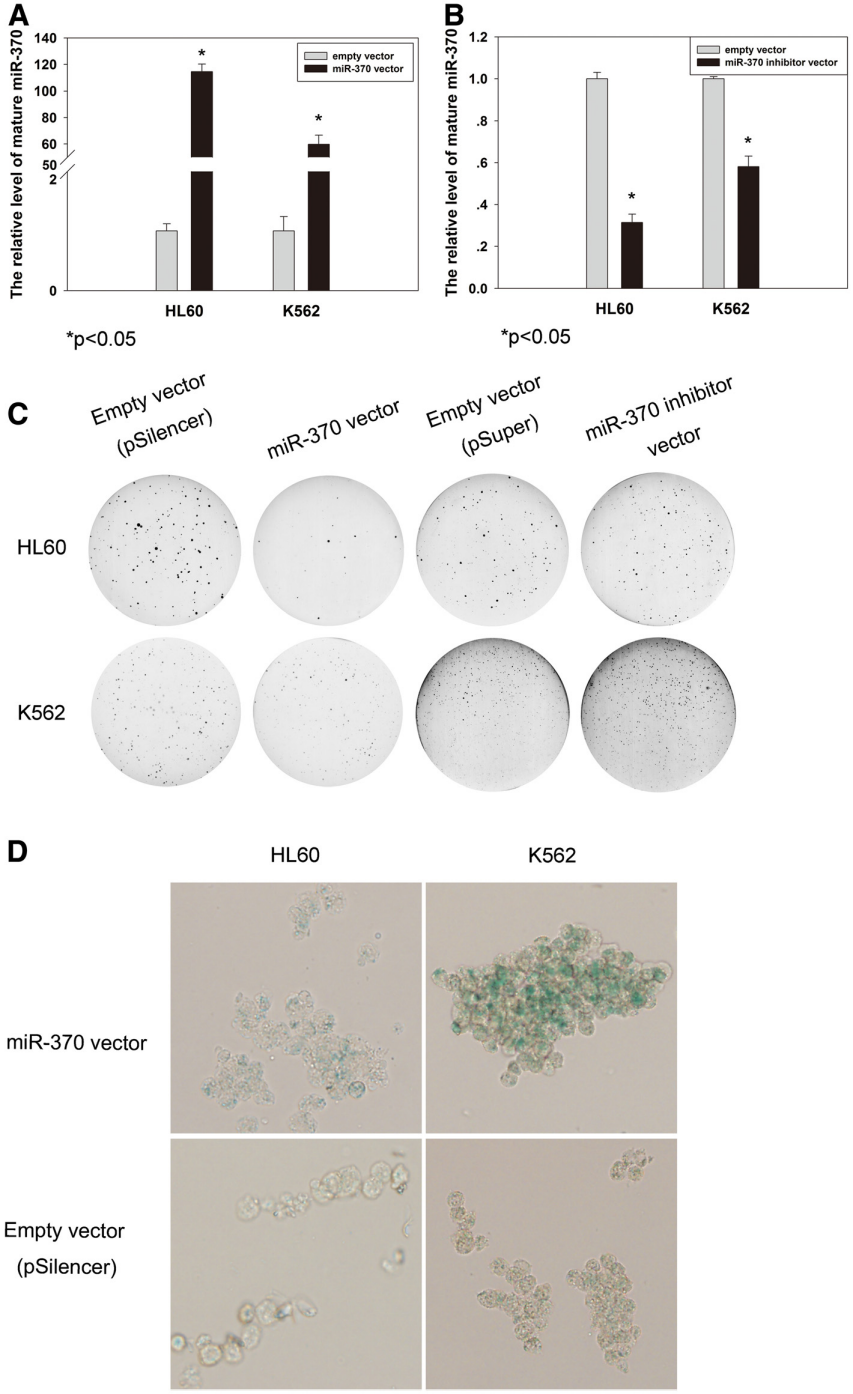
**Suppression of AML cell proliferation and induction of cell senescence by overexpression of miR-370 in vitro while anti-miR-370 expression increases cellular ability of proliferation.** (**A**) *miR-370* expression in AML cell lines and its overexpression using a specific miR-expressing pSilencer vector. The cells were transfected with either miR-370 precursors or with control precursor and harvested after 48 hours. qRT-PCR was applied to detect mature *miR-370* level. (**B**) The depletion of *miR-370* expression by a specific *miR-370*-inhibiting pSuper plasmid. The method was similar as above. (**C**) Alterations in foci formation after different treatments in AML cells. The cells were first treated as above and the efficiently changed *miR-370* expression was verified in (A) and (B). For clonogenic assays, 5,000 cells/well (in 6-well plates) were incubated for 14 days and the foci number was counted. (**D**) Senescence onset of *miR-370*-overexpressed AML cells. β-Gal staining was performed and % of positive cells was calculated

We then explored the biological function of miR-370 in leukemic cells. Cells were transfected with precursors to miR-370 and miR-370 inhibitor to enhance and decrease mature miR-370 expression, respectively. Transfection with the miR-370 precursor increased mature miR-370 expression 114.5 ± 5.70: 1 ± 0.12 (p < 0.05) and 59.8 ± 6.90: 1 ± 0.24 (p < 0.05) (pSilencer-miR vs pSilencer) times higher in HL60 and K562 cells, respectively (Figure 2A). Overexpression of miR-370 decreased cell proliferation (Figure 2C and Additional file 1) (pSilencer vs pSilencer-miR: HL60: 88 ± 15 vs 11 ± 4, p < 0.01; K562: 49 ± 5 vs 18 ± 5, p < 0.01). On the other hand, transfection with the miR-370 inhibitor suppressed mature miR-370 expression to 31% ± 0.04 (p < 0.05) and 58% ± 0.05 (p < 0.05) lower in HL60 and K562 cells, respectively (Figure 2B). The decline in miR-370 expression was coupled with enhanced cell proliferation (Figure 2C) (pSuper vs pSuper-miR-inhibitor: HL60: 56 ± 7 vs 72 ± 6, p < 0.05; K562: 66 ± 12 vs 93 ± 7, p < 0.05).

The above result suggests that miR-370 suppresses proliferation of HL60 and K562 cells. We further wanted to define the mechanism behind miR-370 overexpression-mediated proliferation inhibition. We suspected that miR-370 might trigger cellular senescence program. Senescence-associated β-Gal staining, a specific marker for senescent cells [19], was thus performed. A positive β-Gal staining was observed in the two cell lines transfected with miR-370 precursors (Figure 2D) [pSilencer vs pSilencer-miR (% of β-Gal-positive cells): HL60: 3 ± 1 vs 28 ± 3, p < 0.01; K562: 8 ± 3 vs 40 ± 1, p < 0.01;].

### Up-regulation of miR-370 expression mediated by 5-Aza-CdR

**Figure 3:**
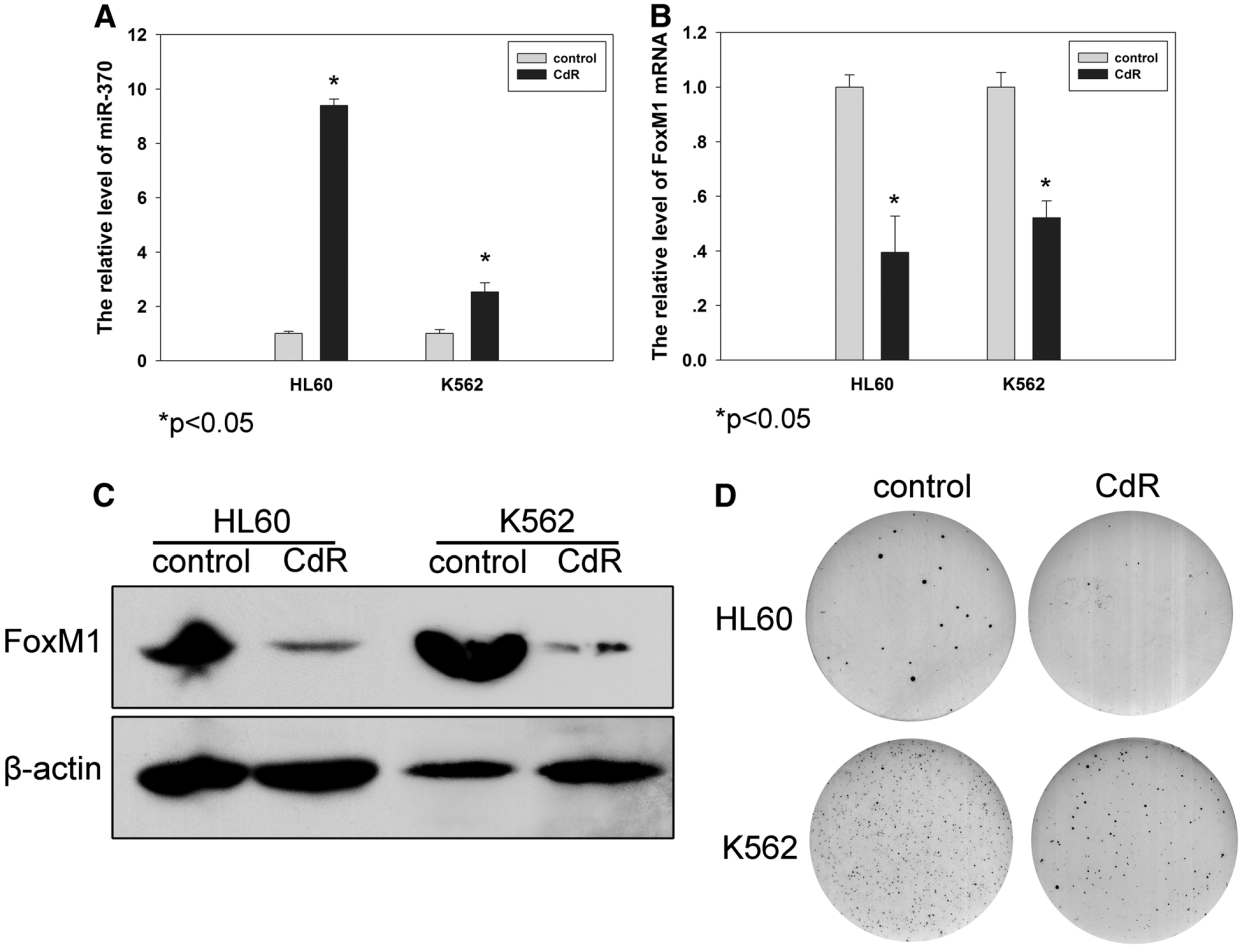
**Expression of miR-370 in AML cells (HL60 and K562) and the effect of 5-aza-2’deoxycytidine (5-Aza-CdR).** AML cells were plated in 6-well plates, treated with 5 μM 5-Aza-CdR or PBS and harvested after 72 hours. The expression of mature miR-370 was assessed using real-time PCR. (**A**) 5-aza-CdR increases the relative expression of miR-370 in AML cells. Data represent mean ± s.e. from three separate determinations. *p < 0.05 compared with respective controls. (**B**) 5-aza-CdR decreases the relative mRNA level of FoxM1 in AML cells. *p < 0.05. (**C**) 5-aza-CdR decreases the protein level of FoxM1 in AML cells. (**D**) Diminished foci formation of 5-aza-CdR treated AML cells

DNA methylation is an epigenetic modification that regulates gene expression. Aberrant DNA methylation has been implicated in many cancers [20]. Global hypomethylation or aberrant hypermethylation of gene promoter CpG islands result, respectively, in tumor cell genomic instability and gene silencing, particularly of tumor suppressor genes [17]. Interestingly, the chromosomal location of miR-370 on chromosome 14q32.31 has been shown to be regulated by DNA methylation, or deleted by loss of heterozygosity [14,21] or by hypermethylation of an CpG island 200 bp upstream in the mother allele [22]. Treatment with 5 μM 5-aza-CdR, a DNA methylation inhibitor, for 72 hours, substantially (>2.0-fold) and significantly (P < 0.05) increased the expression of miR-370 in both HL60 and K562 cells (Figure 3A) and decreased cell proliferation (Figure 3D) (control vs CdR: HL60: 24 ± 4 vs 7 ± 2, p < 0.01; K562: 152 ± 5 vs 78 ± 5, p < 0.001).

### Identification of FoxM1 as a target for miR-370

**Figure 4:**
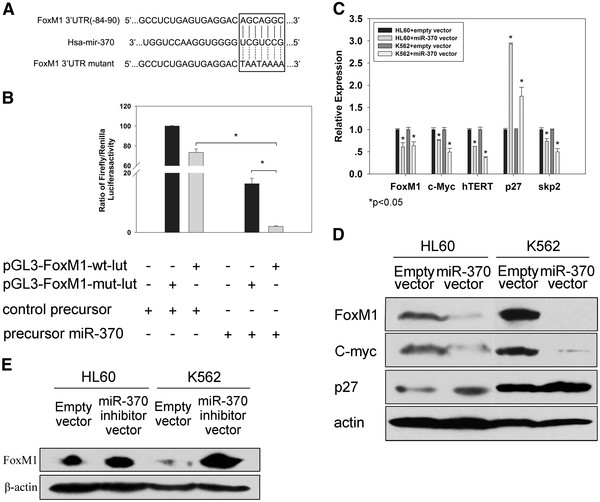
**Identification of *FoxM1 *as a target of miR-370.** (**A**) Predicted site of miR-370 in the *FoxM1* 3’ UTR mRNA. (**B**) Luciferase activities were determined at 24 hours and were normalized by Renilla luciferase activity. K562 cells were plated in 24-well plates, and then transfected with 0.05 μg of a Renilla luciferase expression construct pRL-TK and 0.5 μg of the pGL3-FoxM1-wt-luc or pGL3-FoxM1-mut-luc firefly luciferase expression construct, along with either miR-370 precursor or control precursor. Luciferase assays were performed after 24 hours using the dual luciferase reporter assay system. The expression of firefly luciferase activity was normalized to that of renilla luciferase activity for each sample. A decrease in relative firefly luciferase activity in the presence of miR-370 indicates the presence of a miR-370 modulated target sequence in the 3’-UTR of FoxM1. Data represents mean ± s.e. from three separate experiments. *P < 0.05. (**C**) and (**D**) The expression of FoxM1 and downstream genes were regulated by miR-370 overexpression at both transcription level and translation level. Western blots were performed and sequentially probed with antibodies against FoxM1, c-Myc and p27^kip1^, β-actin was a loading control. Efficient *miR-370* overexpression was verified in Figure 2 (A). (**E**) Increase in FoxM1 protein level after anti-miR-370 treatment. Efficient *miR-370* depletion was verified in Figure 2 (B)

To further elucidate the mechanism by which miR-370 affected cellular senescence and proliferation, we next screened for potential targets of miR-370 using four target prediction programs with different algorithms: DIANA-MicroT [23], TargetScan [24], Miranda [25] and PicTar [26]. All potential targets predicted by more than one of these programs were identified. We selected the forkhead box M1 (FoxM1) for further study because of its well-characterized role in tumor biology. The FoxM1 gene has a 249-bp 3^′^UTR region that presents a 7-mer binding site for miR-370 (Figure 4A).

First, we made the luciferase reporter constructs containing the miR-370 recognition sequence from the 3^′^-UTR of FoxM1 inserted downstream of the luciferase gene. Transfection with miR-370 precursor decreased reporter activity in K562 cells (Figure 4B), which strongly indicates that FoxM1 is a target for miR-370. Next, the studies were repeated with random mutations in the recognition sequence (Figure 4A), which resulted in abolition of the reporter activation by miR-370 precursor (Figure 4B). Finally, we assessed the effect of miR-370 expression on FoxM1 expression. Transfection of HL60 and K562 cells with miR-370 precursor resulted in lower expression of FoxM1 after 48 hours (Figure 4C-D). Concomitant with decreased FoxM1 expression, there was reduction of its downstream target *c-myc* and *skp2* (Figure 4C-D). There was a >2-fold increase in expression of FoxM1 in HL60 and K562 cells after transfection of miR370 inhibitor plasimids (Figure 4E). 5-aza-CdR significantly reduced the expression of FoxM1 in both HL60 and K562 cells (Figure 3 B-C). These changes were similar to those observed with miR-370 overexpression. Taken together, FoxM1 is a target of miR-370.

### Overexpression of FoxM1 in de novo AML patients

**Figure 5:**
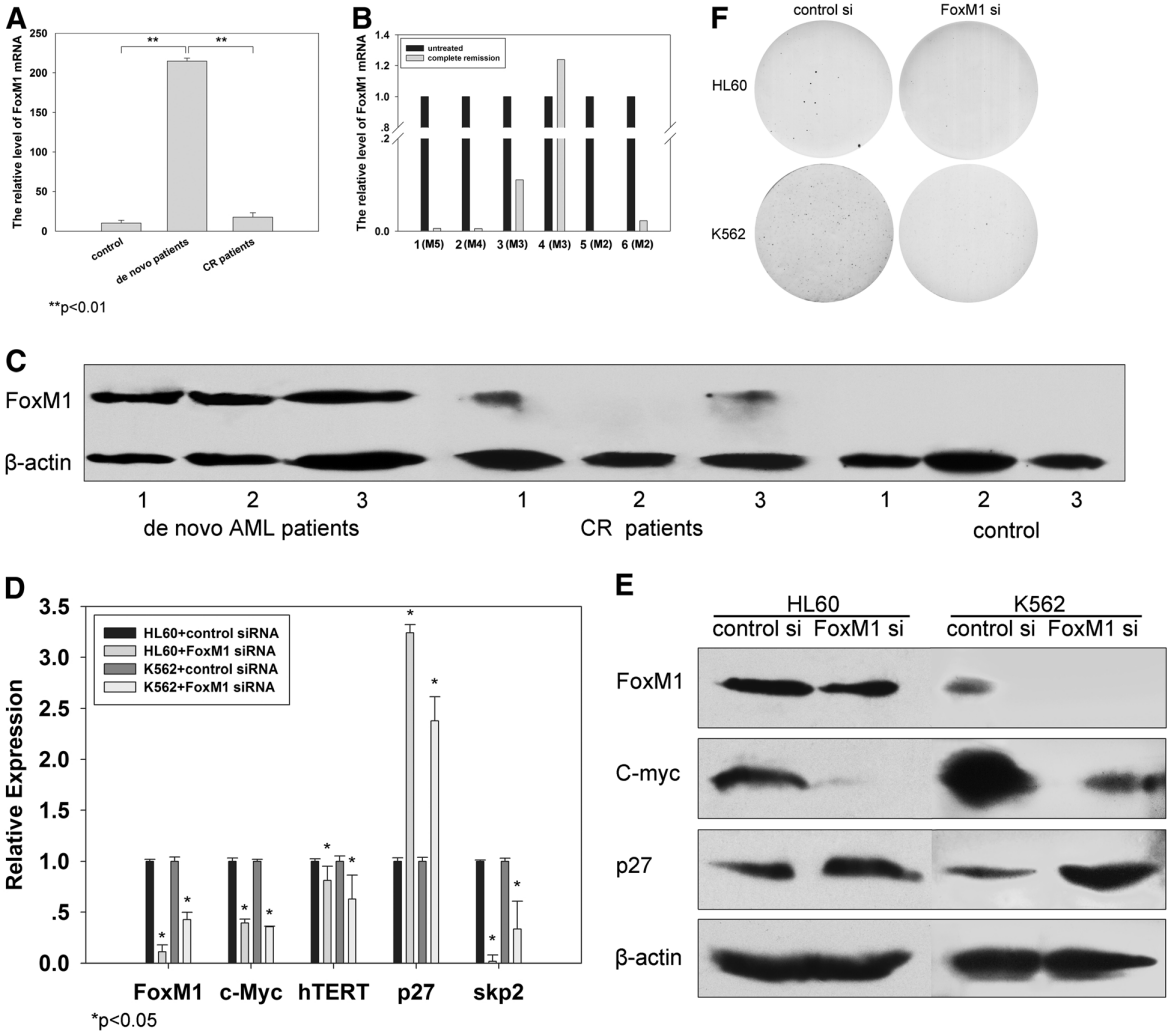
**Overexpression of FoxM1 in de novo AML patients and defective foci formation induced by depletion of FoxM1 in AML cell lines.** (**A**) and (**C**) Expression of FoxM1 mRNA and protein in de novo AML patients, AML patients of 1st CR and controls, were determined using qRT-PCR and Western blot respectively. (**B**) Decrease of FoxM1 expression in six patients after complete remission achievement in induction chemotherapy. (**D**) and (**E**) FoxM1 depletion triggers concomitant down-regulation of c-myc, hTERT and skp2 coupled with the accumulation of the CDK inhibitor p27^kip1^ in AML cells. The cells were transfected with either control or FoxM1 siRNA and harvested after 72 hours. qRT-PCR and western blot were applied to detect mRNA and protein levels, respectively. (**F**) Diminished foci formation of FoxM1-depleted AML cells. The cells were first treated as above and the efficient FoxM1 knock down was verified in (D) and (E)

FoxM1, a master regulator of mitotic gene expression, is required for cell proliferation and its inhibition leads to reduction in anchor-independent growth and tumorigenesis of cancer cells [27]. As we have verified that FoxM1 is a target for miR-370, we then sought to probe its role in AML. The tumor specimens from forty-eight de novo AML patients and forty AML patients in 1st CR and twenty-one healthy controls were analyzed for FoxM1 mRNA expression using qRT-PCR. Patient characteristics are described in Table 1. The FoxM1 transcript level in AML patients was found 21.47-fold higher than that in controls, while following acquisition of CR in the induction chemotherapy, FoxM1 expression level reduced to 1.75 fold of controls. (Figure 5 A, C), which was negatively correlated with miR-370 levels. There was a highly significant difference in FoxM1 expression between AML samples, CR samples and healthy controls (qRT-PCR, One-Way ANOVA, p < 0.01). In six patients, BM samples were available both at diagnosis time prior to treatment and after a complete remission and we found a higher FoxM1 level at diagnosis while a significant decrease in FoxM1 expression after CR except one sample (Figure 5B). There was no clear association between the presence of FoxM1 mRNA and age, gender, tumor burden or FAB subtypes (data not shown). Moreover, BM materials from 8 de novo AML patients, 8 patients in 1st CR and 5 healthy controls randomly chosen from our AML patient pool were used to determine the mRNA expression level of *c-Myc*, *hTERT*, *p27*^*kip1*^ and *skp2*, all of which were the target genes of FoxM1, using real-time PCR method. We found that the transcript levels of *c-Myc*, *hTERT* and *skp2* in AML patients were respectively 9.64, 3.76 and 3.14-fold higher than those in controls, while following acquisition of CR, all of them reduced almost to the same levels of controls. On the contrary, the expression level of *p27*^*kip1*^ in AML patients is only 40% of that in controls, while restored after CR (Additional file 2).

**Table 1:** Patient Characteristics

**Characteristic**	**De novo AML patients**	**AML patients in 1**^**st**^**CR**
Patients,no.	48	40
Sex,male/female,no.(%)	31(65)/17(35)	24(60)/16(40)
Median age,y(range)	41(14–74)	41(14–68)
**WBC count (10**^**9**^**/L)**		
Median	46.3	6.3
Range	0.8-354.9	2.6-10.2
**Hemoglobin (g/dL)**		
Median	7.7	11.8
Range	4.1-13.2	8.3-16.1
**Platelet count (10**^**9**^**/L)**		
Median	58	243.3
Range	2-319	76-384
**BM blasts (%)**		
Median	69.1	2.1
Range	(24–96)	(0–5)
**FAB subtype,n/(%)**		
M2	9/(19)	6/(15)
M3	11/(23)	20/(50)
M4	11/(23)	8/(20)
M5	15/(31)	5/(12.5)
M6	2/(4)	1/(2.5)

### Defective foci formation by depletion of FoxM1 in AML cell lines

Then, we employed the FoxM1-specific siRNA to knock down FoxM1 expression in AML cell lines and the cells were then assessed for their clonogenic capacity. Efficient silence of FoxM1 expression in these cells was verified using qRT-PCR and Western blot analyses (Figure 5 D-E). Compared to the control cells, the FoxM1-knocked down cells exhibited significantly diminished foci formation (Figure 5F) (Controls vs FoxM1 siRNA: HL60: 19 ± 3 vs 11 ± 2, p < 0.05; K562: 33 ± 5 vs 5 ± 2, p = 0.001). Following FoxM1 depletion, its target genes *c-myc, skp2* and *hTERT* were also down-regulated and coupled with the accumulation of the CDK inhibitor *p27*^*kip1*^ (Figure 5 D-E).

Collectively, the *FoxM1* gene is aberrantly activated in AML and is required for sustained proliferation of the cancer cells.

## Discussion

In the present study, we explored the expression and role of miR-370 in AML. Our findings show a down-regulation of miR-370 in blasts from patients with de novo AML. Moreover, we identified FoxM1 as a target for miR-370 and restored expression of miR-370 reduced the level of FoxM1. In hematological malignancies a link with miRNA was initially described in chronic lymphocytic leukemia (CLL) by Calin et al. [28]. A cluster of two miRNAs, miR-15a, and miR-16, was found to be located within the deleted region at 13q14, and down-regulated in the majority (70%) of CLL samples. Notably, miR-15a and miR-16-1 down-regulation contribute to malignant transformation by up-regulating BCL2 [29]. Recently, involvement of miRNA in AML has been documented identifying that miRNA expression profiles are AML subtype-specific and suggesting a pathogenetic role of miRNA in AML. For example, Mi and colleagues [30] showed distinct miRNA signatures between acute lymphoblastic leukemias (ALLs) and AMLs. In that study, patients with AML could be separated from those with ALL on the basis of 21 up-regulated and 6 down-regulated miRNAs, among which four of them (let-7b, miR-128a, miR-128b and miR-223) were the most discriminatory [31]. According to Amanda Dixon-McIver, compared with other major cytogenetic subgroups of AML, APLs bearing the t(15;17) translocation were characterised by the up regulation of 7 miRNAs transcribed from genes located at the 14q32 region. The set includes miR-127, miR-154, miR-154*, miR-299, miR-323, miR-368, and miR-370. Two other groups reported independently that miR-155 was up-regulated in AML patients with FLT3-ITD, suggesting that this miRNA contributes to the highly proliferative phenotype of this molecular subset of AML [31-33]. In the present study, we haven’t got the conclusion that the expression level of miR-370 is with AML subtype-specificity, which may be due to the limited number of primary AML patients enrolled in our study.

DNA methylation is an epigenetic modification that can regulate gene expression. Aberrant DNA methylation has been implicated in many cancers [21]. miR-370 and many other miRNAs are organised in clusters together on chromosome 14q32 [34]. This miRNA cluster acts as imprinted non-coding RNA (ncRNA) genes, which are mono-allelically expressed in a parent-of-origin manner (their expression is restricted to either the maternal or the paternal allele). Interestingly, as for this miRNA cluster, which is of maternal origin, its imprinted expression is regulated by an intergenic differentially methylated region (DMR) located ~ 200 kb upstream from the miRNA cluster [24]. Hypermethylation of DMR causes silence of this miRNA cluster, including miR-370. It has been suggested that miRNAs in this region act as tumour repressor genes and that changes in the methylation status of their promoters could trigger cancer development [35]. For instance, miR-127 has been shown to be down regulated or silenced in cancer cells, whose expression is correlated with the methylation and acetylation status of its promoter. Inhibition of methylation and histone deacetylation in these cancer cells causes over expression of miR-127 and related down regulation of the target BCL6, a bona fide protooncogene [36]. We hypothesize that miR-370 also acts as a tumor suppressor in AML, as in papillary thyroid carcinoma, colorectal cancer and malignant cholangiocytes. The comparison of the leukemia samples with healthy controls highlighted the differential expression of miR-370. Following the treatment with 5-aza-2^′^-deoxycytidine, there is a significant enrichment for miR-370 in AML cell lines, which indicated that hypermethylation may contribute to reduction of miR-370.

Cancer therapy has traditionally relied on cytotoxic treatment strategies on the assumption that complete cellular destruction of tumors optimizes the potential for patient survival. Although these approaches produce complete cell death within a tumor, they also can cause severe side effects in patients [37]. Recently, a promising approach to preventing continued tumor growth is therapy-induced senescence (TIS) [38]. Senescent cells remain viable and metabolically active but are permanently growth arrested [39]. Evidence has recently accumulated that cellular senescence is a potent barrier to cancer development. Our results indeed demonstrated that senescence occurred in most of AML cells treated with miR-370 overexpressing plasmid, which was concomitant with their diminished clonogenic capacity. Given a critical role for senescence induction in tumor suppression and therapeutic efficacy of cancer treatment, the present findings have important biological and clinical implications. All these results suggest that downregulation of miR-370 may be another mechanism involved in the pathology of AML and therefore, could be used as a diagnostic marker and therapeutic target in AML.

We have also analyzed the correlation between miR-370 expression and FoxM1 mRNA expression in 48 de novo AML samples. Consistent with the cell line data, FoxM1 was enriched in the primary blasts mRNAs that inversely correlated to miR-370 expression levels. This result was also observed at the protein level in a few primary AML samples. However, further studies using large numbers of primary AML samples will be needed to confirm this interaction.

## Conclusion

We demonstrate that miR-370 is a tumor suppressive factor by targeting multiple critical oncogenic pathways. Restoring miR-370 expression downmodulates FoxM1, induces senescence, and dampens cell growth in AML cells, thereby suggesting miRNA-based therapy as a novel approach to increase response in AML.

## Materials and methods

### Patients and bone marrow samples

Forty-eight newly diagnosed AML patients (31 male and 17 female; median age at diagnosis 41; range 14–74), forty AML patients in 1^st^ complete remission (CR) and twenty-one healthy controls were enrolled in this study. Diagnosis of AML was established according to clinical presentation and morphologic criteria of the French-American-British (FAB) Classification. The study was approved by the local ethics committee. Patients’ BM samples were collected between April 2008 and September 2011 at the Department of Hematology, Qilu Hospital, Shandong University, Jinan, China. Mononuclear cells were isolated using Ficoll-Hypaque density gradient centrifugation, and then stored at −80°C until use. All patients and healthy controls were tested for miR-370 and FoxM1 mRNA levels in their BM cells. Among those AML patients, six were analyzed for miR-370 and FoxM1 levels in their bone marrow samples at both diagnosis and complete remission.

### Cell lines and culture conditions

Human AML cell lines HL60 and K562 were cultured at 37°C, 95% air, 5% CO_2_ in RPMI 1640 containing 10% heat-inactivated fetal bovine serum (FBS; Gibco, Carlsbad, CA, USA), 100 μg/mL penicillin, and 50 μg/mL streptomycin. To assess 5-aza-CdR (Sigma, Santa Clara, CA, USA) effects, cells were grown on 6-well plates, treated with 5 μM 5-aza-CdR or cold phosphate-buffered saline (PBS) controls for 72 h at 37°C, and then harvested for isolation of total mRNA or protein.

### RNA extraction and quantitative real-time PCR

Total cellular RNA in BM samples and in cells with different treatments was extracted using the Trizol (Invitrogen, Carlsbad, CA, USA) according to the manufacturer’s protocol. cDNA was synthesized using random primers (N6) (Fermentas, St. Leon-Rot, Germany) and MMLV reverse transcriptase. The PCR primers used in the study were as follows: sequences specific for FoxM1 mRNA: 5^′^-TGCAGCTAGGATGTGAATCTTC-3^′^ (Forward) and 5^′^-GGAGCCCAGTCCATCAGAACT-3^′^ (Reverse). Skp2: 5^′^-GGACCTATCGAACTCAGTTAT-3^′^ (Forward) and 5^′^-CAGCCACCTGTACATGCTTT-3^′^ (Reverse); p27^kip^: 5^′^-ATGTCAAACGTGCGAGTGTCTAA-3^′^ (Forward) and 5^′^-TTACGTTTGACGTCTTCTGAGG-3^′^ (Reverse); hTERT: 5^′^-CGGAAGAGTGTCTGGAGCAA-3^′^ (Forward) and 5^′^-GGATGAAGCGGAGTCTGGA-3^′^ (Reverse); c-MYC: 5^′^-TACCCTCTCAACGACAGCAGCTCGCCCAACTCCT-3^′^ (Forward) and 5^′^-TCTTGACATTCTCCTCGGTGTCCGAGGACCT-3^′^ (Reverse). The above primer pairs cross intron/exon boundaries; thus, the resultant PCR products do not represent genomic DNA contamination. β-actin expression was used as a control for RNA loading and RT efficiency and amplified. Quantitative real-time polymerase chain reaction (qRT-PCR) was carried out in an ABI7000 sequence detector (Applied Biosystems, Foster City, CA, USA).

### TaqMan qRT-PCR miRNA analysis

Quantification of mature miRNAs was performed using qRT-PCR with the TaqMan miRNA assay kit (Applied Biosystems, Foster City, CA, USA) according to manufacturer’s instruction. Briefly, 10 ng of total RNA was reverse-transcribed (RT) with specific primers, subsequently 1.5 μL of RT product was used as template for real-time PCR. All real-time experiments were performed in triplicate. Data were normalized by the expression of small nuclear RNA (snRNA) U6 and expressed either as relative expression (2^-ΔCt^) or as fold change relative to control (2^-ΔΔCt^).

### Western blot

Total cellular proteins were extracted from cultured cells or BM samples. Proteins were resolved by SDS-PAGE and transferred to a nitrocellulose membrane. The membranes were probed with the specific antibodies against FoxM1, p27, c-MYC (Santa Cruz Biotechnologies, Santa Cruz, CA, USA), followed by anti-mouse or rabbit horseradish peroxidase–conjugated IgG and developed with the enhanced chemiluminescence method (ECL). β-actin served as a loading control.

### miR-expressing and miR-inhibiting plasmids

To generate the miR-expressing pSilencer3.1-H1 neo vector, a fragment of 212 base pairs (bp) corresponding to the desired miRNA and the surrounding sequences was amplified from human genomic sequence, adding a BamHI site and a HindIII site to the 5^′^ and 3^′^ ends respectively, using the polymerase chain reaction (PCR) with primers sense: 5^′^ AAGGGATCCTACTTGAGGGATGGGCGATA 3^′^ and antisense 5^′^ TCAAAGCTTCCCGAGCTCTGGTGTTAGAC 3^′^. We included a large portion of miRNA surrounding sequence in the attempt to allow correct processing of the miRNA to its mature form and to induce overexpression while preserving a physiologic mechanism of miRNA production. miR-370 inhibitor sequences were synthesized as DNA oligonucleotides; after annealing, were sticking ended and subcloned into a pSuper vector.

### Transfection

Cells were incubated in 6-well plates (3.0× 10^5^/well and 1.0× 10^5^/well, respectively) overnight and were then transfected with plasmid or siRNA using Lipofectamine 2000 (Invitrogen, Carlsbad, CA, USA) according to the manufacturer’s protocol. Chemical modified Stealth small interfering RNA (siRNA) targeting FoxM1 and control siRNA were bought from Invitrogen. The sequence for the FoxM1 siRNA was 5^′^-GACAACUGUCAAGUGUACCACUCUU-3^′^.

### Soft agar colony formation assay

HL60 and K562 cells were resuspended in DMEM (Gibco, Carlsbad, CA, USA) containing 20% heat-inactivated fetal bovine serum (FBS; Gibco, Carlsbad, CA, USA) with equal amount of either 0.3% agar (HL60 cells) or 0.5% agar (K562 cells), and plated in 6-well plates at 5,000 per well on top of a 2 mL precast semisolid 1% agar underlayer as described previously [40]. The number of colonies with more than 50 cells was counted after two weeks.

### Senescence-associated β-Galactosidase (SA -β-Gal) staining

SA -β-Gal staining was done as described [19,41]. Briefly, the cells grown in 6-well plates were transfected with pSilencer or pSilencer-miR-370. After 7 days, the cells were rinsed with PBS once, fixed in 3% of formaldehyde for 15 min, and incubated with freshly prepared SA -β-Gal staining solution at 37°C overnight.

### Luciferase reporter vector

The precursor to miR-370 was synthesized and cloned in pSilencer. Firefly luciferase reporter vectors with the intact putative miR-370 recognition sequence from the 3^′^-UTR of FoxM1 (pGL3-FoxM1-wt-3^′^-UTR) or with random mutations (pGL3-FoxM1-mut-3^′^-UTR) cloned downstream of the firefly luciferase gene were constructed. Wild-type and mutant inserts were confirmed by sequencing. For the 3^′^UTR-luciferase assays, cells were co-transfected with 0.5 μg pGL3-FoxM1-wt or mut-3^′^-UTR construct, 4 μg of pSilencer or pSilencer-miR and 0.05 μg pRL-TK Renilla luciferase expression construct using Lipofectamine 2000. Luciferase assays were performed 24 h after transfection using the Dual Luciferase Reporter Assay system (Promega, Madison, Wisconsin, USA).

### Statistical analyses

The difference in miR-370 and FoxM1 mRNA expression among different patient groups as detected using qRT-PCR was analyzed using One-Way ANOVA. The comparison of foci numbers, β-Gal-positive cells, luciferase activity and miR-370, FoxM1, c-myc, hTERT, p27, skp2 mRNA expression after different treatments was made using a Student’s t-test. All the tests were two-tailed and computed using SPSS11.5 software. Results are depicted as the mean ± standard error of the mean. P values < 0.05 were defined as statistical significance.

## Competing interests

The authors declare no competing financial interests.

## Authors’ contributions

JZ, CC and XZ designed the study; XZ, JZ, BL, MZ, YZ and TH performed the research; XZ ,JZ, LW, JJ, and CC analyzed and interpreted data; and XZ, JZ and CC wrote the paper. All authors read and approved the final manuscript.

## Supplementary Material

Additional file 1 **Proliferation curve of HL60 cell line (A) and K562 cell line (B) after transfection with miR-370-expressing plasmid or the control pSilencer vector.** 1 × 10^5^/ml cells were plated in 6-well plates just before the transfection. The number of viable cells was counted at 24 h and 48 h points using trypan blue. Data represents mean ± s.e. from three separate experiments. **p < 0.01 Student’s t-test. Click here for file

Additional file 2 ***c-Myc, hTERT, p27 ***^***kip1 ***^**and *skp2 *expression by qRT-PCR in 8 de novo AML patients, 8 AML patients of 1st CR and 5 healthy controls.** The transcript levels of *c-Myc, hTERT* and *skp2* in AML patients were found respectively 9.64, 3.76 and 3.14-fold higher than those in controls, while following acquisition of CR in the induction chemotherapy, all of them reduced almost to the same levels of controls. On the contrary, the expression level of p27^kip1^ in AML patients is only 40% of that in controls, while restored after CR. *p < 0.05 One-Way ANOVA Click here for file

## References

[B1] Estey E Döhner H Acute myeloid leukaemia Lancet 2006 368 1894 1907 10.1016/S0140-6736(06)69780-8 17126723

[B2] Estey EH Treatment of acute myeloid leukemia Haematologica 2009 94 10 16 10.3324/haematol.2008.001263 19118375 PMC2625419

[B3] Altuvia Y Landgraf P Lithwick G Elefant N Pfeffer S Aravin A Brownstein MJ Tuschl T Margalit H Clustering and conservation patterns of human microRNAs Nucleic Acids Res 2005 33 2697 2706 10.1093/nar/gki567 15891114 PMC1110742

[B4] Bartel DP MicroRNAs: genomics, biogenesis, mechanism, and function Cell 2004 116 281 297 10.1016/S0092-8674(04)00045-5 14744438

[B5] He L Hannon GJ MicroRNAs: small RNAs with a big role in gene regulation Nat Rev Genet 2004 5 522 531 10.1038/nrg1379 15211354

[B6] Cheng AM Byrom MW Shelton J Ford LP Antisense inhibition of human miRNAs and indications for an involvement of miRNA in cell growth and apoptosis Nucleic Acids Res 2005 33 1290 1297 10.1093/nar/gki200 15741182 PMC552951

[B7] Karp X Ambros V Encountering microRNAs in cell fate signaling Science 2005 310 1288 1289 10.1126/science.1121566 16311325

[B8] Xu P Guo M Hay BA MicroRNAs and the regulation of cell death Trends Genet 2004 20 617 624 10.1016/j.tig.2004.09.010 15522457

[B9] Chen C-Z Li L Lodish HF Bartel DP MicroRNAs modulate hematopoietic lineage differentiation Science 2004 303 83 86 10.1126/science.1091903 14657504

[B10] Poy MN Eliasson L Krutzfeldt J Kuwajima S Ma X MacDonald PE Pfeffer S Tuschl T Rajewsky N Rorsman P Stoffel M A pancreatic islet-specific microRNA regulates insulin secretion Nature 2004 432 226 230 10.1038/nature03076 15538371

[B11] Calin GA Sevignani C Dumitru CD Hyslop T Noch E Yendamuri S Shimizu M Rattan S Bullrich F Negrini M Croce CM Human microRNA genes are frequently located at fragile sites and genomic regions involved in cancers Proc Natl Acad Sci U S A 2003 101 2999 3004 14973191 10.1073/pnas.0307323101 PMC365734

[B12] Rooij E Sutherland LB Liu N Williams AH McAnally J Gerard RD Richardson JA Olson EN A signature pattern of stress-responsive microRNAs that can evoke cardiac hypertrophy and heart failure Proc Natl Acad Sci U S A 2006 103 18255 18260 10.1073/pnas.0608791103 17108080 PMC1838739

[B13] Calin GA Croce CM MicroRNA signatures in human cancers Nat Rev Cancer 2006 6 857 866 10.1038/nrc1997 17060945

[B14] Starczynowski DT Morin R McPherson A Lam J Chari R Wegrzyn J Kuchenbauer F Hirst M Tohyama K Humphries RK Genome-wide identification of human microRNAs located in leukemia-associated genomic alterations Blood 2011 117 595 607 10.1182/blood-2010-03-277012 20962326

[B15] Baltimore D Boldin MP O’Connell RM Rao DS Taganov KD MicroRNAs: new regulators of immune cell development and function Nat Immunol 2008 9 839 845 10.1038/ni.f.209 18645592

[B16] Marcucci G Mrózek K Radmacher MD Garzon R Bloomfield CD The prognostic and functional role of microRNAs in acute myeloid leukemia Blood 2011 117 1121 1129 10.1182/blood-2010-09-191312 21045193 PMC3056468

[B17] Bandrés E Cubedo E Agirre X Malumbres R Zárate R Ramirez N Abajo A Navarro A Moreno I Monzó M García-Foncillas J Identification by real-time PCR of 13 mature microRNAs differentially expressed in colorectal cancer and non-tumoral tissues Mol Cancer 2006 5 29 39 16854228 10.1186/1476-4598-5-29 PMC1550420

[B18] Meng F Wehbe-Janek H Henson R Smith H Patel T Epigenetic regulation of microRNA-370 by interleukin-6 in malignant human cholangiocytes Oncogene 2008 27 378 386 10.1038/sj.onc.1210648 17621267

[B19] Dimri GP Lee X Basile G Acosta M Sco G Roskelley C Medrano EE Linskensi M Rubeljii I Pereira-Smithii O A biomarker that identifies senescent human cells in culture and in aging skin in vivo Proc Natl Acad Sci U S A 1995 92 9363 9367 10.1073/pnas.92.20.9363 7568133 PMC40985

[B20] Baylin SB DNA methylation and gene silencing in cancer Nat Clin Pract Oncol 2005 2 S4 S11 16341240 10.1038/ncponc0354

[B21] Calin G Croce C MicroRNAs and chromosomal abnormalities in cancer cells Oncogene 2006 25 6202 6210 10.1038/sj.onc.1209910 17028600

[B22] Hln R Jrm C Non-coding RNAs in imprinted gene clusters Biol Cell 2008 100 3 149 166 10.1042/BC20070126 18271756

[B23] Kiriakidou M Nelson PT Kouranov A Fitziev P Bouyioukos C Mourelatos Z Hatzigeorgiou A A combined computational-experimental approach predicts human microRNA targets Genes Dev 2004 18 1165 1178 10.1101/gad.1184704 15131085 PMC415641

[B24] Lewis BP Burge CB Bartel DP Conserved seed pairing, often flanked by adenosines, indicates that thousands of human genes are microRNA targets Cell 2005 120 15 20 10.1016/j.cell.2004.12.035 15652477

[B25] John B Enright AJ Aravin A Tuschl T Sander C Marks DS Human microRNA targets PLoS Biol 2004 2 1862 1879 10.1371/journal.pbio.0020363 PMC521178 15502875

[B26] Krek A Grün D Poy MN Wolf R Rosenberg L Epstein EJ MacMenamin P Piedade I Gunsalus KC Stoffel M Rajewsky N Combinatorial microRNA target predictions Nat Genet 2005 37 495 500 10.1038/ng1536 15806104

[B27] Wierstra I Alves J FOXM1, a typical proliferation-associated transcription factor Biol Chem 2007 388 1257 1274 18020943 10.1515/BC.2007.159

[B28] Calin GA Dumitru CD Shimizu M Bichi R Zupo S Noch E Aldler H Rattan S Keating M Rai K Frequent deletions and down-regulation of micro-RNA genes miR15 and miR16 at 13q14 in chronic lymphocytic leukemia Proc Natl Acad Sci U S A 2002 99 15524 15529 10.1073/pnas.242606799 12434020 PMC137750

[B29] Cimmino A Calin GA Fabbri M Iorio MV Ferracin M Shimizu M Wojcik SE Aqeilan RI Zupo S Dono M miR-15 and miR-16 induce apoptosis by targeting BCL2 Proc Natl Acad Sci U S A 2005 102 13944 13949 10.1073/pnas.0506654102 16166262 PMC1236577

[B30] Mi S Lu J Sun M Li Z Zhang H Neilly MB Wang Y Qian Z Jin J Zhang Y MicroRNA expression signatures accurately discriminate acute lymphoblastic leukemia from acute myeloid leukemia Proc Natl Acad Sci U S A 2007 104 19971 19976 10.1073/pnas.0709313104 18056805 PMC2148407

[B31] Garzon R Volinia S Liu C-G Fernandez-Cymering C Palumbo T Bloomfield Andreeff M Croce CM Flomenberg Marcucci G MicroRNA signatures associated with cytogenetics and prognosis in acute myeloid leukemia Blood 2008 111 3183 3189 10.1182/blood-2007-07-098749 18187662 PMC2265455

[B32] Garzon R Garofalo M Martelli MP Briesewitz R Wang L Fernandez-Cymering C Volinia S Liu C-G Schnittger S Haferlach T Distinctive microRNA signature of acute myeloid leukemia bearing cytoplasmic mutated nucleophosmin Proc Natl Acad Sci U S A 2008 105 3945 3950 10.1073/pnas.0800135105 18308931 PMC2268779

[B33] Jongen-Lavrencic M Sun SM Dijkstra MK Valk PJM Löwenberg B MicroRNA expression profiling in relation to the genetic heterogeneity of acute myeloid leukemia Blood 2008 111 5078 5085 10.1182/blood-2008-01-133355 18337557

[B34] Seitz H Royo H Bortolin M-L Lin S-P Ferguson-Smith AC Cavaillé J A large imprinted microRNA gene cluster at the mouse Dlk1-Gtl2 domain Genome Res 2004 14 1741 1748 10.1101/gr.2743304 15310658 PMC515320

[B35] Saito Y Liang G Egger G Friedman JM Chuang JC Coetzee GA Jones PA Specific activation of microRNA-127 with downregulation of the proto-oncogene BCL6 by chromatin-modifying drugs in human cancer cells Cancer Cell 2006 9 435 443 10.1016/j.ccr.2006.04.020 16766263

[B36] Phan RT Dalla-Favera R The BCL6 proto-oncogene suppresses p53 expression in germinal-centre B cells Nature 2004 432 635 639 10.1038/nature03147 15577913

[B37] Ewald JA Desotelle JA Wilding G Jarrard DF Therapy-induced senescence in cancer J Natl Cancer Inst 2010 102 1536 1546 10.1093/jnci/djq364 20858887 PMC2957429

[B38] Roninson IB Tumor cell senescence in cancer treatment Cancer Res 2003 63 2705 2715 12782571

[B39] Campisi J Fagagna FA Cellular senescence: when bad things happen to good cells Nat Rev Mol Cell Biol 2007 8 729 740 17667954 10.1038/nrm2233

[B40] Li X-N Parikh S Shu Q Jung H-L Chow C-W Perlaky L Leung H-CE SJ Blaney S Lau CC Phenylbutyrate and phenylacetate induce differentiation and inhibit proliferation of human medulloblastoma cells Clin Cancer Res 2004 10 1150 1159 10.1158/1078-0432.CCR-0747-3 14871995

[B41] Lindvall C Hou M Komurasaki T Zheng C Henriksson M Sedivy JM Björkholm M Teh BT Nordenskjöld M Xu D Molecular characterization of human telomerase reverse transcriptase-immortalized human fibroblasts by gene expression profiling: activation of the epiregulin gene Cancer Res 2003 63 1743 1747 12702554

