# Supplementary figures and images for: The tumor suppressive role of miRNA-370 by targeting FoxM1 in acute myeloid leukemia
Source: Mol Cancer. 2012 Aug 17;11:56. doi: 10.1186/1476-4598-11-56 (PMC3533721; doi:10.1186/1476-4598-11-56)

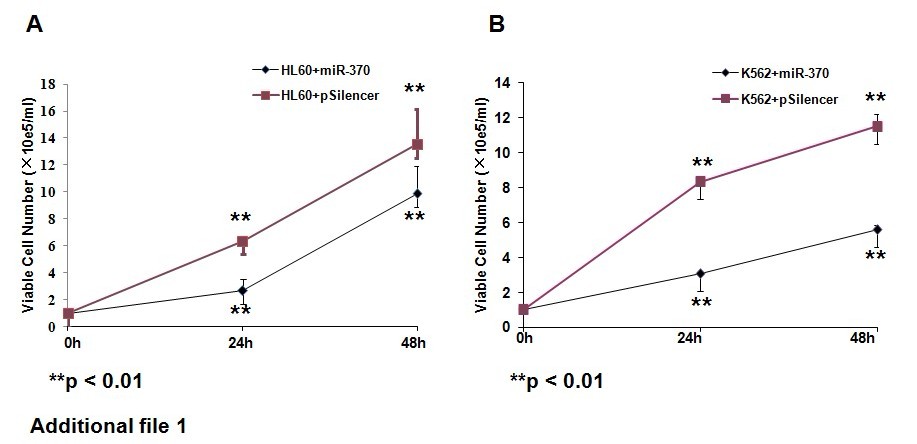

Supplement: Additional file 1 — Proliferation curve of HL60 cell line (A) and K562 cell line (B) after transfection with miR-370-expressing plasmid or the control pSilencer vector. 1 × 10^5^/ml cells were plated in 6-well plates just before the transfection. The number of viable cells was counted at 24 h and 48 h points using trypan blue. Data represents mean ± s.e. from three separate experiments. **p < 0.01 Student’s t-test. [file 1476-4598-11-56-S1.jpeg]

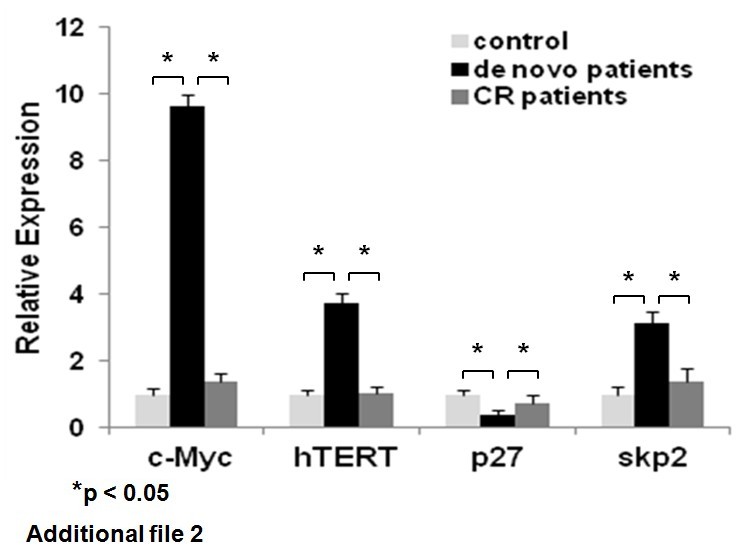

Supplement: Additional file 2 — c-Myc, hTERT, p27 kip1 and skp2 expression by qRT-PCR in 8 de novo AML patients, 8 AML patients of 1st CR and 5 healthy controls. The transcript levels of c-Myc, hTERT and skp2 in AML patients were found respectively 9.64, 3.76 and 3.14-fold higher than those in controls, while following acquisition of CR in the induction chemotherapy, all of them reduced almost to the same levels of controls. On the contrary, the expression level of p27kip1 in AML patients is only 40% of that in controls, while restored after CR. *p < 0.05 One-Way ANOVA [file 1476-4598-11-56-S2.jpeg]
